# ExCAPE-DB: an integrated large scale dataset facilitating Big Data analysis in chemogenomics

**DOI:** 10.1186/s13321-017-0203-5

**Published:** 2017-03-07

**Authors:** Jiangming Sun, Nina Jeliazkova, Vladimir Chupakhin, Jose-Felipe Golib-Dzib, Ola Engkvist, Lars Carlsson, Jörg Wegner, Hugo Ceulemans, Ivan Georgiev, Vedrin Jeliazkov, Nikolay Kochev, Thomas J. Ashby, Hongming Chen

**Affiliations:** 1 Discovery Sciences, Innovative Medicines and Early Development Biotech Unit, AstraZeneca R&D Gothenburg, 43183 Mölndal, Sweden; 2 Ideaconsult Ltd., 4. Angel Kanchev Str., 1000 Sofia, Bulgaria; 3 Computational Biology, Discovery Sciences, Janssen Pharmaceutica NV, Turnhoutseweg 30, 2349 Beerse, Belgium; 4 Computational Biology, Discovery Sciences, Janssen Cilag SA, Calle Río Jarama, 71A, 45007 Toledo, Spain; 5 Department of Analytical Chemistry and Computer Chemistry, University of Plovdiv, Plovdiv, Bulgaria; 6 Imec vzw, Kappeldreef 75, 3001 Louvain, Belgium

**Keywords:** Big Data, Bioactivity, Chemogenomics, Chemical structure, Molecular fingerprints, Search engine, QSAR

## Abstract

Chemogenomics data generally refers to the activity data of chemical compounds on an array of protein targets and represents an important source of information for building *in silico* target prediction models. The increasing volume of chemogenomics data offers exciting opportunities to build models based on Big Data. Preparing a high quality data set is a vital step in realizing this goal and this work aims to compile such a comprehensive chemogenomics dataset. This dataset comprises over 70 million SAR data points from publicly available databases (PubChem and ChEMBL) including structure, target information and activity annotations. Our aspiration is to create a useful chemogenomics resource reflecting industry-scale data not only for building predictive models of *in silico* polypharmacology and off-target effects but also for the validation of cheminformatics approaches in general.

**Electronic supplementary material:**

The online version of this article (doi:10.1186/s13321-017-0203-5) contains supplementary material, which is available to authorized users.

## Background

In pharmacology, “Big Data” on protein activity and gene expression perturbations has grown rapidly over the past decade thanks to the tremendous development of proteomics and genome sequencing technology [1, 2]. Similarly there has also been a remarkable increase in the amount of available compound structure and activity relation (SAR) data, contributed mainly by the development of high throughput screening (HTS) technologies and combinatorial chemistry for compound synthesis [3]. These SAR data points represent an important resource for chemogenomics modelling, a computational strategy in drug discovery that investigates an interaction of a large set of compounds (one or more libraries) against families of functionally related proteins [4].

Frequently, the “Big Data” in chemogenomics refers to large databases recording the bioactivity annotation of chemical compounds against different protein targets. Databases such as PubChem [5], BindingDB [6], and ChEMBL [7] are examples of large public domain repositories of this kind of information. PubChem is a well-known public repository for storing small molecules and their biological activity data [5, 8]. It was originally started as a central repository of HTS experiments for the National Institute of Health (USA) Molecular Libraries Program, but nowadays also incorporates data from other sources. ChEMBL contains data that was manually extracted from numerous peer reviewed journal articles, as do WOMBAT [9], BindingDB [6], and CARLSBAD [10]. Similarly, commercial databases, such as SciFinder [11], GOSTAR [12] and Reaxys [13] have accumulated a large amount of data from publications as well as patents. Besides these sources, large pharmaceutical companies maintain their own data collections originating from in-house HTS screening campaigns and drug discovery projects.

This data serves as a valuable source for building *in silico* models for predicting polypharmacology and off-target effects, and benchmarking the prediction performance and computation speed of machine-learning algorithms. The aforementioned publicly available databases have been widely used in numerous cheminformatics studies [14–16]. However, the curated data are quite heterogeneous [17] and lack a standard way for annotating biological endpoints, mode of action and target identifier. There is an urgent need to create an integrated data source with a standardized form for chemical structure, activity annotation and target identifier, covering as large a chemical and target space as possible. There are also irregularities within databases: the public screening data in PubChem, especially the inactive data points, are spread in different assay entries uploaded by data providers from around world and cannot be directly compared without processing. This makes curating SAR data for quantitative structure–activity relationship (QSAR) modeling very tedious. An example of work to synthesize the curated and uncurated data is Mervin et al. [15], where a dataset with ChEMBL active compounds and Pubchem inactive compounds was constructed, including inactive compounds for homologous proteins. However, the dataset can only be accessed as a plain text file, not as a searchable database.

In this work, by combining active and inactive compounds from both PubChem and ChEMBL, we created an integrated dataset for cheminformatics modeling purposes to be used in the ExCAPE [18] (Exascale Compound Activity Prediction Engine) Horizon 2020 project. ExCAPE-DB, a searchable open access database, was established for sharing the dataset. It will serve as a data hub for giving researchers around world easy access to a publicly available standardized chemogenomics dataset, with the data and accompanying software available under open licenses.

## Dataset curation

The standardized ChEMBL20 data from an in-house database ChemistryConnect [3] was extracted and PubChem data was downloaded in January 2016 from the PubChem website (https://pubchem.ncbi.nlm.nih.gov/) using the REST API. Both data sources are heterogeneous. Data cleaning and standardisation procedures were applied in preparing both chemical structures and bioactivity data.

### Chemical structure standardisation

Standardisation of PubChem and ChEMBL chemical structures was performed with ambitcli version 3.0.2. The ambitcli tool is part of the AMBIT cheminformatics platform [19–21] and relies on The Chemistry Development Kit library 1.5 [22, 23]. It includes a number of chemical structure processing options (fragment splitting, isotope removal, handling implicit hydrogens, stereochemistry, InChI [24] generation, SMILES [25] generation and structure transformation via SMIRKS [26], tautomer generation and neutralisation etc.). The details of the structure processing procedure can be found in Additional file 1. All standardisation rules were aligned between Janssen Pharmaceutica, AstraZeneca and IDEAConsult to reflect industry standards and implemented in open source software (https://doi.org/10.5281/zenodo.173560).

### Bioactivity data standardisation

**Fig. 1:**
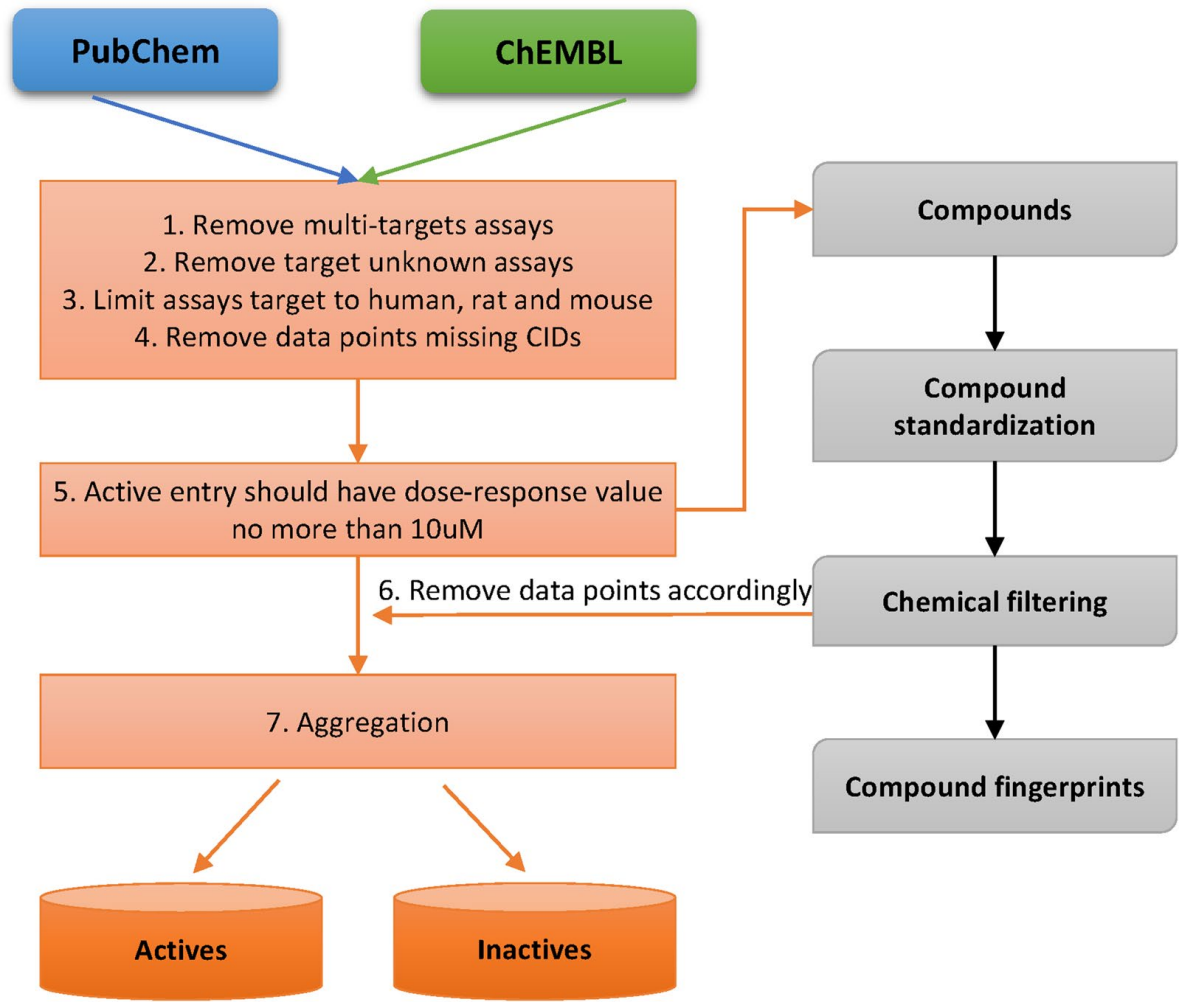
Workflow for data preparation

The processing protocol for extracting and standardizing bioactivity data is shown in Fig. 1. First, bioassays were restricted to only those comprising a single target; the black box (target unknown) or multi-target assays were excluded. 58,235 and 92,147 single targets containing concentration response (CR) type assays (confirmatory type in PubChem) remained in PubChem and ChEMBL, respectively. The assay target was further limited to human, rat and mouse species, and data points missing a compound identifier (CID) were removed. For those filtered assays, active compounds whose dose–response value was equal to or lower than 10 μM were kept as active entries and others were removed. Inactive compounds in CR assays were kept as inactive entries. Compounds that were labelled as inactive in PubChem screening assays (assays run with a single concentration) were also kept as inactive records.

The chemical structure identifiers (InChI, InChIKey and SMILES) generated from the standardized compound structures (as explained above) were joined with the compounds obtained after the filtering procedure.

The compound set was further filtered by the following physicochemical properties: organic filters (compounds without metal atoms), molecular weight (MW) <1000 Da, and a number of heavy atoms (HEV) >12. This was done to remove small or inorganic compounds not representative for modelling the chemical space relevant for a normal drug discovery project. This is a much more generous rule than the Lipinski rule-of-five [27], but the aim was to keep as much useful chemical information as possible while still removing some non-drug like compounds. Finally, fingerprint descriptors were generated for all remaining compounds. So far JCompoundMapper (JCM) [28], CDK circular fingerprint descriptors and signature descriptors [29] were generated respectively. For circular fingerprint and signature calculation, the maximum topological radius for fragment generation was set to 3.

From each data source, various attributes were read and converted into controlled vocabularies. The most important of these are target (Entrez ID), activity value, mode of action, assay type and assay technology etc. The underlying data sources contain activity data with various result types; the results were unified as best possible to make them comparable across tests (and data sources) irrespective of the original result type. The selected compatible dose–response result types are listed in Additional file 2: Table S1. Generally, the end-point name of a concentration related assay (e.g. IC50, units in µM) should match one of the keywords in this list. In the case when a compound has multiple activity data records for the same target, the records are aggregated so that one compound only has one record per target and the best (maximal) potency was chosen as the final aggregated value for a compound–target pair. The AMBIT generated InChIKey from the standardisation procedure was used as the molecular identifier to identify duplicate structures in the data aggregation. Finally, targets which have <20 active compounds were removed from the final dataset.

Entrez ID [30], gene symbol [31–33] and gene orthologue were collected as information for the target. The gene symbol was converted from Entrez ID with the gene2accession table [34] provided by National Center for Biotechnology Information (NCBI). Gene orthologues was included from the orthologue table [34] from NCBI.

### Database and web interface

The ExCAPE-DB is built based on the AMBIT database and web application [19], enhanced with a free text search engine (Apache Solr [35]). An instance of the AMBIT web application (ambit2.war) was installed and the chemical structures were imported. This enables chemistry-aware search (similarity, substructure) and depiction, all exposed via a REST API and the web interface provided by the web application itself. The bioactivity data, consisting of compound related information (e.g. target activity label and InChIKey) and target related information (e.g. Entrez IDs and official gene symbols), is imported into an Apache Solr collection (http://lucene.apache.org/solr/) and exposed through the Solr REST API. The open source JavaScript client library jToxKit (https://github.com/ideaconsult/jToxKit) is used to interact with the AMBIT REST API and the Solr REST API. A dedicated JavaScript web interface was developed for ExCAPE-DB, integrating the chemical search, as well as the free text and faceted search functionality for biological activities.

**Fig. 2:**
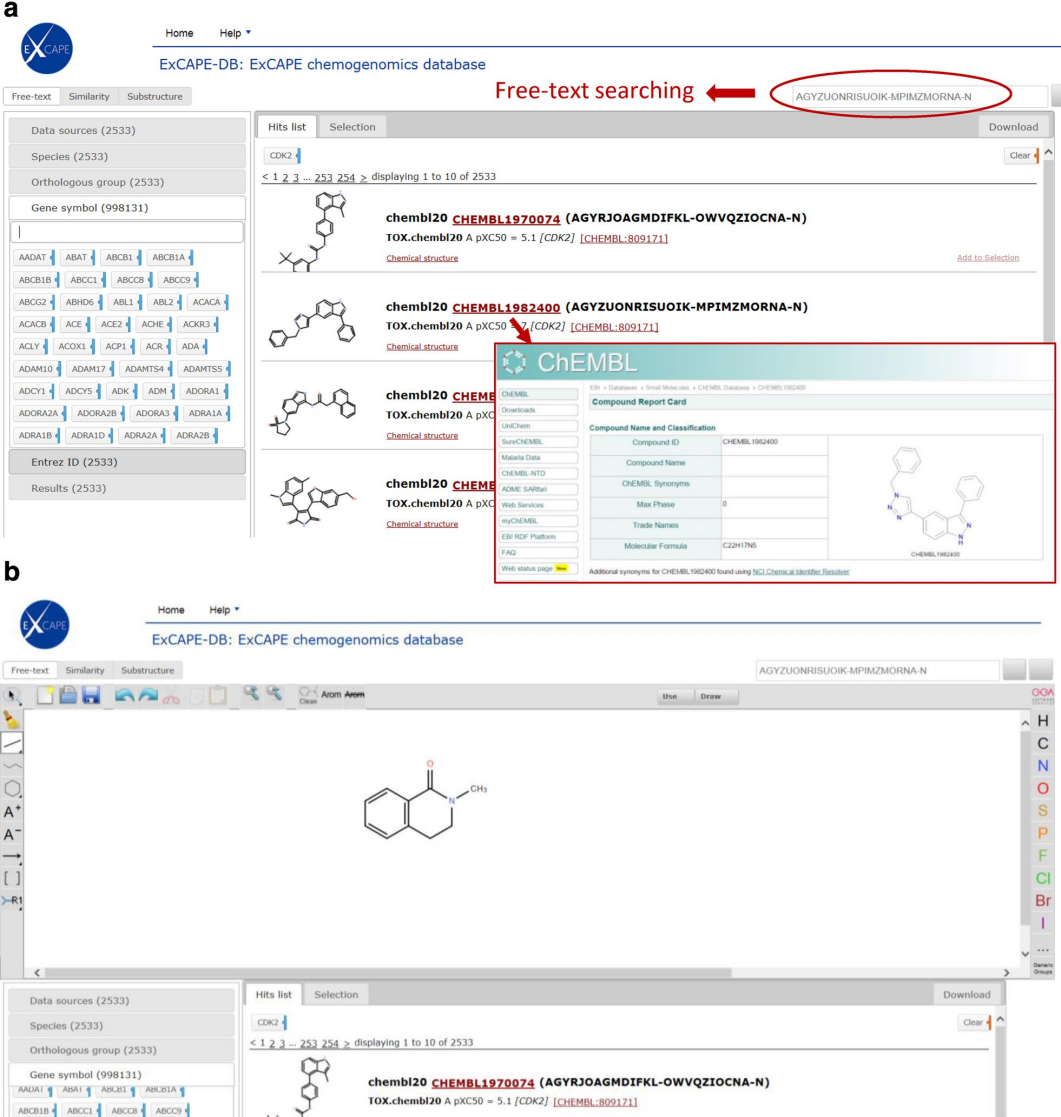
Browsing the ExCAPE-DB web interface. **a** Searching the database via gene symbol or free-text. The original compound information is linked to from the result page. **b** Searching the database via substructure search

The ExCAPE-DB is available online (https://solr.ideaconsult.net/search/excape/) and a screenshot of the web browser interface is shown in Fig. 2a. The dataset can be searched both by target name and CID. For target based searches, the Entrez ID, gene symbol, gene orthologous group and target species can be used for subsetting datasets. For compound searches, a user can choose to input the InChIKey or specify a CID (SMILES, InChI or IUPAC chemical name) for doing free-text search or use the embedded structure editor for doing substructure or similarity search (Fig. 2b). It is also possible to follow a link to the original ChEMBL or PubChem page of the specific compound from the search result. The download tab on the web page provides several download options. The “Filtered entries” download option allows the downloading of all of the current search result. For downloading specific entries, it is possible to include “Add to selection” links and compile a subset of selected entries, which will be available for download as “Selected entries”. A static link for downloading the entire ExCAPE-DB dataset is available at the download tab. The dataset is also uploaded to the Zenodo.org repository and available for download from there as doi:10.5281/zenodo.173258.

## Discussion

**Fig. 3:**
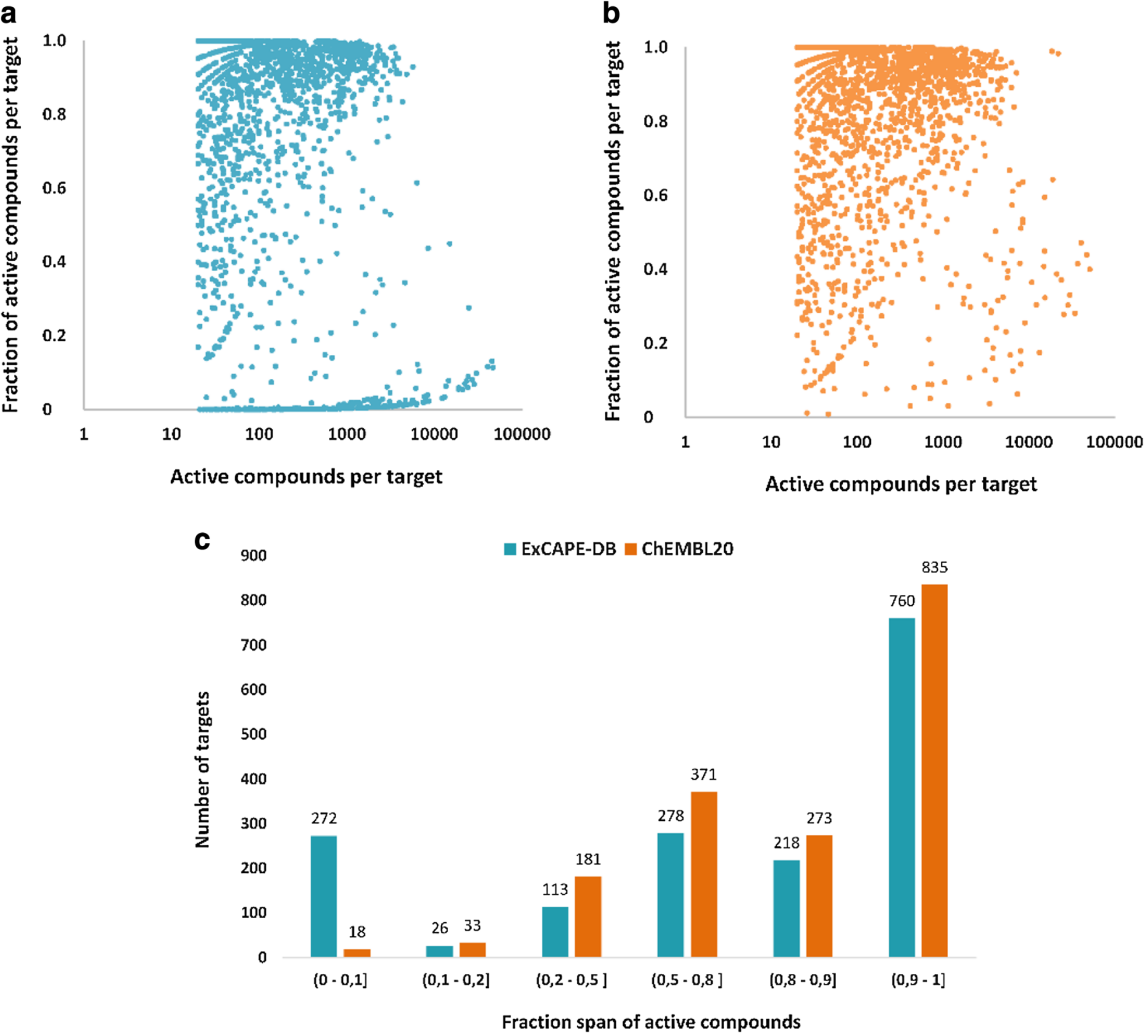
Composition of active compounds in the dataset. The distribution of active compounds among the targets in **a** ExCAPE-DB, **b** ChEMBL part of ExCAPE-DB and **c** the fraction span of actives in both datasets. We note that the ChEMBL dataset is shown here before the filtering and aggregation process and contains only single-target assays. Active compounds should have a pXC50 no less than 5 and only targets with at least 20 active compounds were considered

The dataset composition is described in Table 1. In total there are 998,131 unique compounds and 70,850,163 SAR data points. These SAR data points cover 1667 targets (Additional file 3: Table S2). It constitutes a curated large scale chemogenomics set freely available in the public domain under the Creative Commons Attribution Share-Alike 4.0 license. The dataset is useful for building QSAR models for predicting activity against one or more specific targets for novel compounds and will also serve as a benchmark dataset for evaluating the performance of various machine-learning algorithms, especially multi-target learning algorithms. The distribution of active compounds of ExCAPE-DB and ChEMBL themselves are shown in Fig. 3. Overall, most targets have far fewer inactive compounds than active compounds, which means that the chemogenomics dataset is highly imbalanced in both the ChEMBL and ExCAPE-DB datasets.

**Table 1:** Public chemogenomics dataset

	ChEMBL	PubChem	ExCAPE-DB
Actives			
# SAR data points	1,259,338	439,288	1,332,426
# Compounds	566,143	263,119	593,156
Inactives			
# SAR data points	1,530,908	68,948,609	69,517,737
# Compounds	416,655	654,562	719,192
Total			
# SAR data points	2,790,246	69,387,897	70,850,163
# Compounds	710,324	828,317	998,131
# Targets	1644	1588	1667

By adding inactive compounds from PubChem, the ExCAPE-DB has many more targets where the fraction of active compounds is <10% of the total number of compounds. Inclusion of inactive compounds from PubChem better mimics chemogenomics datasets available in the pharmaceutical industry, and it has been shown that inclusion of true inactive compounds results in better models than using random compounds as inactive compounds [15]. A low ratio between active and inactive compounds also reflects better the results of high-throughput screening where the hit rate is usually around 1%.

**Fig. 4:**
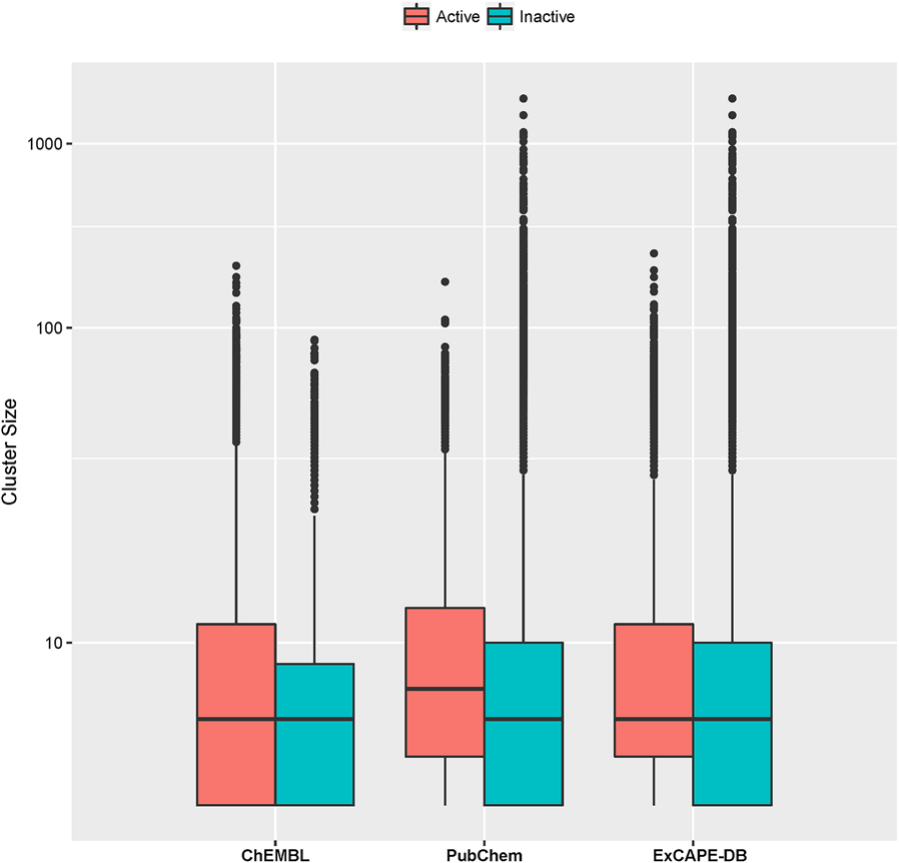
Distribution of cluster size in ExCAPE-DB. Here singletons and small clusters whose size is <4 are excluded from the analysis

A clustering analysis was carried out for ChEMBL, PubChem and ExCAPE-DB compounds (as shown in Table 1) using an in-house program Flush [36] with a default Tanimoto similarity threshold of 0.7 that was calculated based on Foyfi fingerprints [37]. The distribution of cluster size for active compounds and inactive compounds is shown in Fig. 4. Here the singletons and small clusters whose size is <4 are excluded to give a better comparison. It can be seen that the cluster sizes of ChEMBL active and inactive compounds are very similar, while Pubchem active compounds tend to have a larger cluster size than the inactive compounds and hence they are less diverse than the inactive compounds. This is probably due to the fact that ChEMBL is composed of a series of analogue compounds, while the inactive compounds from screening campaigns in PubChem are more likely to be structurally diverse compounds. The SAR data is provided as is, but the underlying differences on structural diversity between active and inactive compounds should be considered when using ExCAPE-DB data for modelling.

**Fig. 5:**
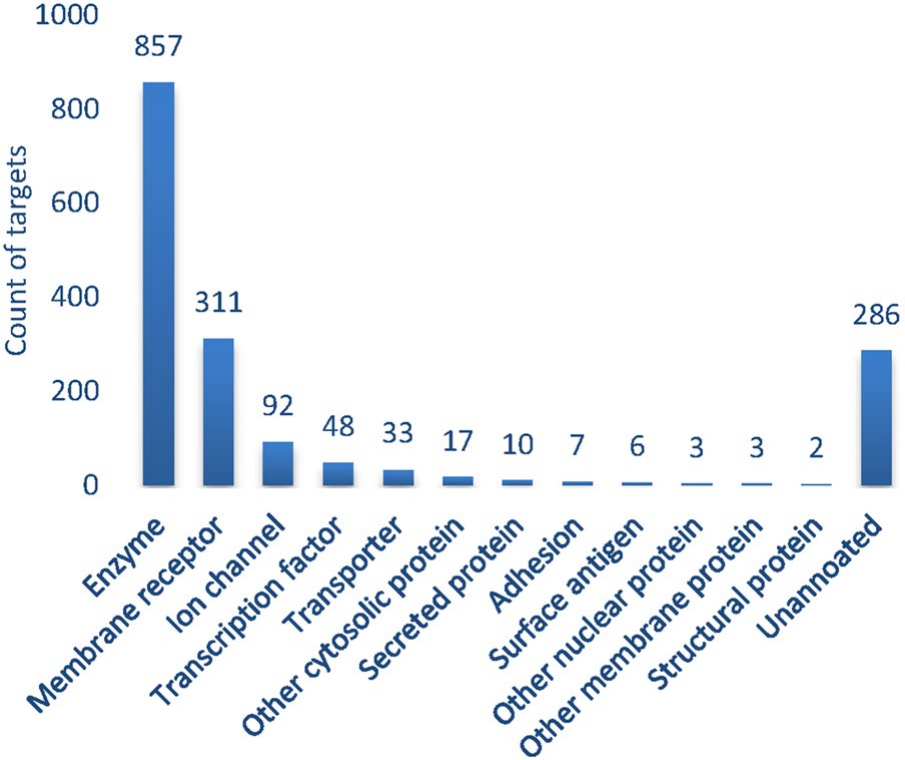
Target family distribution in the dataset

**Fig. 6:**
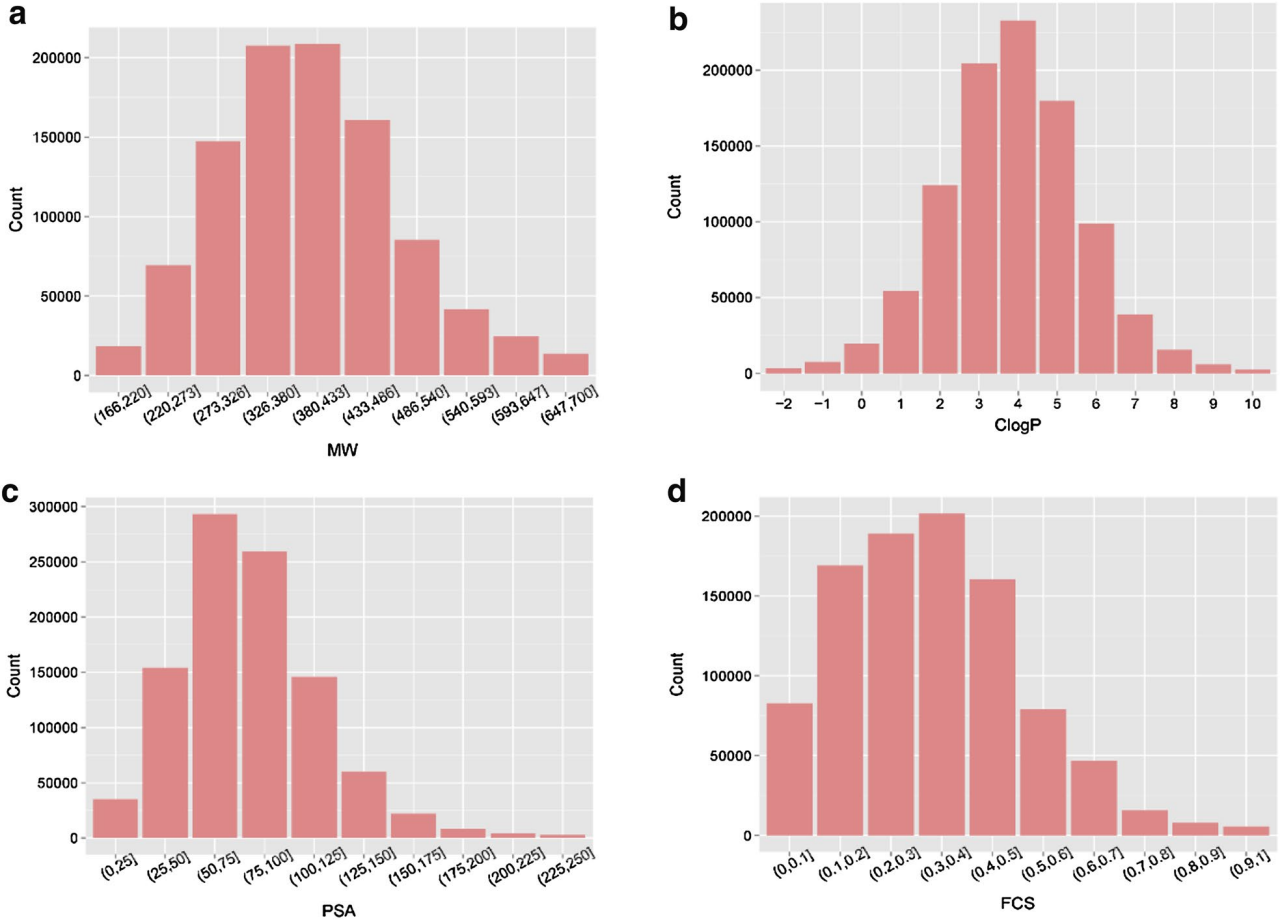
The physicochemical property distribution. **a** Molecular weight (MW), **b** calculated value of lipophilic efficiency (ClogP), **c** polar surface area (PSA) and **d** fraction of *sp*3 carbon (FCS)

The target class distribution across the dataset was also examined. The results are described in Fig. 5 for several major target families. The most common target class is enzymes followed by membrane receptors and then ion channels and transcription factors. The physicochemical property distribution of the dataset is shown in Fig. 6. Figure 6a–d are for MW, ClogP [38] representing calculated lipophilicity, polar surface area (PSA) which represent compounds polarity, and fraction of *sp*3 carbon atoms (F*sp*3) in the compound which is a measure of the “flatness” of a compound [39], respectively. The MW of most compounds is between 220 and 540 Da. ClogP is mainly between 1 and 8. Most compounds have a PSA <150 and F*sp*3 <0.7. In general, these distributions show that most compounds in the dataset fulfil the Lipinski rule-of-five [27] and are considered to be drug like compounds.

As an example of the utility of the generated dataset, 18 targets which have imbalance level varying from 1:10 to 1:1000 (ratio of active/inactive) were chosen for building support vector machine (SVM) models using LIBSVM [40]. Signature descriptors were used as input features. The performance of binary classification is given in Table 2 and model metrics shown are sensitivity, precision, specificity and Cohen’s κ value [41]. The results show that performance as expected varies from case to case and reasonable SVM models can be built even for some severely imbalanced datasets. This validates that the generated data set can be useful for predicting activity for novel compounds and for benchmarking studies.

**Table 2:** Performances of fivefold cross-validation for 18 targets using SVM

Target	Active compounds	Inactive compounds	Ratio (active/inactive compounds)	Sensitivity	Precision	Specificity	κ
PPARA	1955	1465	1.33	0.96	0.94	0.92	0.89
MMP2	2742	2363	1.16	0.96	0.96	0.96	0.92
MAOA	732	733	1.00	0.79	0.80	0.81	0.59
NR1I2	249	1090	0.23	0.82	0.73	0.93	0.72
TMPRSS15	139	724	0.19	0.43	0.54	0.93	0.39
HSD17B10	3410	11,510	0.30	0.41	0.40	0.82	0.23
KDM4E	3938	35,059	0.11	0.22	0.29	0.94	0.18
LMNA	14,533	171,164	0.09	0.49	0.13	0.72	0.10
TDP1	23,133	276,782	0.08	0.76	0.38	0.90	0.45
TARDBP	12,193	387,934	0.03	0.22	0.08	0.92	0.08
ALOX15	1932	69,362	0.03	0.49	0.12	0.90	0.16
BRCA1	8619	363,912	0.02	0.72	0.20	0.93	0.29
DRD2	4613	343,076	0.01	0.96	0.93	1.00	0.94
GSK3B	3334	300,186	0.01	0.85	0.72	1.00	0.78
JAK2	2158	213,915	0.01	0.85	0.81	1.00	0.83
POLK	773	389,418	0.002	0.55	0.17	0.99	0.26
FEN1	1050	381,575	0.003	0.35	0.03	0.96	0.04
HDAC3	369	311,425	0.001	0.98	0.76	1.00	0.86

## Conclusion

ExCAPE-DB is a large public chemogenomics dataset based on the PubChem and ChEMBL databases, and large scale standardisation (including tautomerization) of chemical structures using open source cheminformatics software was performed in data curation. Comprehensive compound related information such as target activity label, fingerprint based descriptors and InChIKey, and target related information such as Entrez IDs and official gene symbols were collected and are easily accessible in the publicly available database. The active labels were determined based on their dose–response data to make sure the data quality is as high as possible. This ‘Big Data’ set covers large number of targets reported in the literature and can be used for building holistic multi-target QSAR models for target prediction. The data set will be used as a comprehensive benchmark set to evaluate the performance of various machine-learning algorithms in the ExCAPE project. To the best of our knowledge, this is first attempt to build such a large scale and searchable open access database for QSAR modelling.

## Additional files

**Additional file 1.** The protocol for structure standardisation.

**Additional file 2: Table S1.** The list of selected activity types in the PubChem.

**Additional file 3: Table S2.** The list of targets in the final dataset.
