# Erratum to: ExCAPE-DB: an integrated large scale dataset facilitating Big Data analysis in chemogenomics

**DOI:** 10.1186/s13321-017-0222-2

**Published:** 2017-06-14

**Authors:** Jiangming Sun, Nina Jeliazkova, Vladimir Chupakhin, Jose-Felipe Golib-Dzib, Ola Engkvist, Lars Carlsson, Jörg Wegner, Hugo Ceulemans, Ivan Georgiev, Vedrin Jeliazkov, Nikolay Kochev, Thomas J. Ashby, Hongming Chen

**Affiliations:** 1 Discovery Sciences, Innovative Medicines and Early Development Biotech Unit, AstraZeneca R&D Gothenburg, 43183 Mölndal, Sweden; 2 Ideaconsult Ltd., 4. Angel Kanchev Str., 1000 Sofia, Bulgaria; 3 Computational Biology, Discovery Sciences, Janssen Pharmaceutica NV, Turnhoutseweg 30, 2349 Beerse, Belgium; 4 Computational Biology, Discovery Sciences, Janssen Cilag SA, Calle Río Jarama, 71A, 45007 Toledo, Spain; 5 Department of Analytical Chemistry and Computer Chemistry, University of Plovdiv, Plovdiv, Bulgaria; 6 Imec vzw, Kappeldreef 75, 3001 Louvain, Belgium

## Erratum to: J Cheminform (2017) 9:17 DOI 10.1186/s13321-017-0203-5

After publication of this work [1], it was noticed that the author’s name was spelt incorrectly. His name should be spelt as: Vladimir Chupakhin. The ‘h’ was missing from his surname.

The publisher apologises for these errors.

The original article has been updated.
