# Supplementary material for: ExCAPE-DB: an integrated large scale dataset facilitating Big Data analysis in chemogenomics
Source: J Cheminform. 2017 Mar 7;9:17. doi: 10.1186/s13321-017-0203-5 (PMC5340785; doi:10.1186/s13321-017-0203-5)
Supplement: Supplementary file 1 — Additional file 1. The protocol for structure standardisation. [file 13321_2017_203_MOESM1_ESM.docx]

**Additional file 1**

**The protocol for structure standardisation**

PubChem chemical structures were retrieved from PubChem ftp site [1] as SDF files. ChEMBL structures were retrieved as one SDF file [2]. Standardisation was performed with ambitcli version 3.0.2 (build 7433) [3]. The ambitcli tool is part of the AMBIT cheminformatics platform [4-6] and relies on The Chemistry Development Kit library 1.5 [7-8]. It includes a number of chemical structure processing options (fragments splitting, isotopes removal, handling implicit hydrogens, stereochemistry, InChI generation, SMILES generation, structure transformation via SMIRKS, tautomers generation, neutralisation).

The structure standardisation was performed in four steps, as follows:

1) Read original PubChem SDF files, generate SMILES, InChI and InChIKey from the connection table, write output in tabular TXT format with a reduced number of columns that correspond to desired properties.

The ambitcli tool was executed with the following options in this first step (step-01):

java -Xmx1536m -jar ambitcli-3.0.2-7433.jar -a standardize -m post -d page=0 -d pagesize=-1 –d tautomers=false -d tag_tokeep=PUBCHEM_CID,PUBCHEM_OPENEYE_ISO_SMILES -d smilescanonical=false –d smiles=true -d inchi=true -i <input>.sdf -o step-01/<output>_step-01.txt > step-01/logs/<output>_step-01.log 2>&1

where <input> and <output> correspond to an original chunk of PubChem records.

2) Read output files from step-01, perform standardisation, write output in tabular TXT format. The ambitcli tool was executed with the following options in the second step (step-02):

java -Xmx1536m -jar ambitcli-3.0.2-7433.jar -a standardize -m post -d page=0 -d pagesize=-1 –d tag_smiles=AMBIT_SMILES -d tag_inchi=AMBIT_InChI -d tag_inchikey=AMBIT_InChIKey –d tautomers=true -d splitfragments=true -d implicith=true -d smilescanonical=false -d smiles=true -d inchi=true -d neutralise=true -d isotopes=true -d tag_tokeep=PUBCHEM_CID -i step-01/<input>_step-01.txt -o step-02/<output>_step-01_step-02.txt > step-02/logs/<output>_step-01_step-02.log 2>&1

where <input> and <output> correspond to an original chunk of PubChem records.

3) Read output files from step-02 and calculate fingerprints. The ambitcli tool was executed with the following options in the third step (step-03):

java -Xmx1536m -jar ambitcli-3.0.2-7433.jar -a fingerprint -m post -d page=0 -d pagesize=-1 –d fpclass=CircularFingerprinter -d tag_tokeep=AMBIT_InChIKey -d inputtag_smiles=AMBIT_SMILES –d inputtag_inchikey=AMBIT_InChIKey -d inputtag_inchi=AMBIT_InChI -d write_count=true -i step-02/<input>_step-01_step-02.txt -o step-03/<output>_step-01_step-02_step-03.txt > step-03/logs/<output>_step-01_step-02_step-03.log 2>&1

4) Read output files from step-02 and step-03 and merge them in aggregate TXT tables (one master file and three associated fingerprint files).

The processing for ChEMBL follows similar steps. The standardized structures are further used in the compilation of the ExCAPE chemogenomics dataset.

**Reference**

http://ftp.ncbi.nih.gov/pubchem/Compound/CURRENT-Full/SDF/

http://ftp.ebi.ac.uk/pub/databases/chembl/ChEMBLdb/releases/chembl_20/chembl_20.sdf.gz

https://sourceforge.net/projects/ambit/files/Ambit2/AMBIT%20applications/ambitcli/ambitcli-3.0.2/

Jeliazkova N, Jeliazkov V. AMBIT RESTful web services: An implementation of the OpenTox application programming interface. J. Cheminform*.* 2011;3:18.

Jeliazkova N, Kochev N. AMBIT-SMARTS: Efficient searching of chemical structures and fragments. Mol. Inform. 2011;30:707.

Kochev N, Paskaleva V, Jeliazkova N. AMBIT-Tautomer: An open source tool for tautomer generation. Mol. Inform. 2013;32:1.

Steinbeck C, Han Y, Kuhn S, Horlacher O, Luttmann E, Willighagen EL. The Chemistry Development Kit (CDK): an open-source Java library for Chemo- and Bioinformatics. J. Chem. Inf. Comput. Sci. 2003;43:493.

Steinbeck C, Hoppe C, Kuhn S, Floris M, Guha R, Willighagen EL. Recent Developments of the Chemistry Development Kit (CDK) - An Open-Source Java Library for Chemo- and Bioinformatics. Curr. Pharm. Des. 2006;12:2111.
